# Preclinical Development and Production of Virus-Like Particles As Vaccine Candidates for Hepatitis C

**DOI:** 10.3389/fmicb.2017.02413

**Published:** 2017-12-05

**Authors:** Makutiro Ghislain Masavuli, Danushka K. Wijesundara, Joseph Torresi, Eric J. Gowans, Branka Grubor-Bauk

**Affiliations:** ^1^Virology Laboratory, Basil Hetzel Institute for Translational Medicine, Discipline of Surgery, University of Adelaide, Adelaide, SA, Australia; ^2^Department of Microbiology and Immunology, The Peter Doherty Institute for Infection and Immunity, University of Melbourne, Parkville, VIC, Australia

**Keywords:** viral hepatitis, hepatitis C virus, preventative vaccination, virus-like particles, immune response, liver disease

## Abstract

Hepatitis C Virus (HCV) infects 2% of the world’s population and is the leading cause of liver disease and liver transplantation. It poses a serious and growing worldwide public health problem that will only be partially addressed with the introduction of new antiviral therapies. However, these treatments will not prevent re-infection particularly in high risk populations. The introduction of a HCV vaccine has been predicted, using simulation models in a high risk population, to have a significant effect on reducing the incidence of HCV. A vaccine with 50 to 80% efficacy targeted to high-risk intravenous drug users could dramatically reduce HCV incidence in this population. Virus like particles (VLPs) are composed of viral structural proteins which self-assemble into non-infectious particles that lack genetic material and resemble native viruses. Thus, VLPs represent a safe and highly immunogenic vaccine delivery platform able to induce potent adaptive immune responses. Currently, many VLP-based vaccines have entered clinical trials, while licensed VLP vaccines for hepatitis B virus (HBV) and human papilloma virus (HPV) have been in use for many years. The HCV core, E1 and E2 proteins can self-assemble into immunogenic VLPs while inclusion of HCV antigens into heterogenous (chimeric) VLPs is also a promising approach. These VLPs are produced using different expression systems such as bacterial, yeast, mammalian, plant, or insect cells. Here, this paper will review HCV VLP-based vaccines and their immunogenicity in animal models as well as the different expression systems used in their production.

## Introduction

Hepatitis C Virus is an enveloped positive sense single-stranded RNA virus that infects more than 170 million people (∼2% of the world’s population) (Bartenschlager et al., 2011). A majority of the individuals infected with HCV develop chronic hepatitis and a proportion will develop cirrhosis and liver carcinoma. HCV remains the most common reason for liver transplantation worldwide (Bartenschlager et al., 2011).

The introduction of highly effective direct acting antivirals (DAA) designed to inhibit the function of specific non-structural (NS) viral proteins critical to viral replication has resulted in high cure rates in most patients, a shortened duration of treatment and is associated with relatively few side effects. However, the use of DAA is limited by the high cost (up to US$147,000 for 12-week treatment course) (Ahlén et al., 2013; Rosenthal and Graham, 2016). As a consequence, it is estimated that approximately ∼1% of those diagnosed with hepatitis C virus infection are treated annually in Australia (Commonwealth of Australia, 2014), while the global pool of HCV infected persons is increased by 3–4 million new infections each year (Gower et al., 2014). Furthermore, the World Health Organization (WHO) estimates that as of 2015 only 20% of those infected with HCV were aware of their diagnosis and only 7.4% of those diagnosed with HCV were placed on treatment worldwide (World Health Organization, 2017). Consequently, developing a safe, effective and inexpensive HCV vaccine is therefore necessary to control global infection and reduce the financial burden on healthcare systems.

However, the development of an effective HCV vaccine is hindered by several factors including the high genetic variability of the virus genome that gives rise to escape mutants which can evade both cellular and humoral host immune responses. Moreover, currently there are no suitable small animal models available that can mimic HCV infection of humans. Furthermore, humans and chimpanzees who have been previously treated and cured of hepatitis C could be re-infected after re-exposure to the virus (Farci et al., 1992; Bukh et al., 2008). Conversely, there is some optimism that the development of a successful HCV vaccine might be possible because a proportion of infected individuals (15–25%) clear the virus naturally and develop some immunity that is able to reduce the duration of viremia and the viral load in subsequent infections (Prince, 1994; Halliday et al., 2011). An understanding of the antiviral immune responses in these individuals and in the chimpanzee model have elucidated key mechanisms thought to contribute to the control of HCV infection. It was demonstrated that the induction and maintenance of strong CD4++ and CD8+ T cell immune responses against various viral epitopes are associated with the resolution of HCV infection (Forns et al., 2000; Thimme et al., 2001, 2002; Youn et al., 2005). It is now thought that a cell-mediated response alone is not sufficient and that the timely induction of cross-neutralizing antibodies (NAbs) is important for protection and in viral clearance (Farci et al., 1996; Yu et al., 2004; Osburn et al., 2014).

Licensed viral vaccines such as poliovirus, induce NAbs against viral surface proteins to provide protection. However, despite the development of an efficient system for HCV culture (Lindenbach et al., 2005; Wakita et al., 2005; Zhong et al., 2005), it is still not feasible to generate a vaccine based on killed or live-attenuated HCV due to the potential dangers associated with the use of such particles (Forns et al., 2002). These shortcomings have led to the development of experimental vaccines which include DNA vaccines, recombinant (non-pathogenic) vectors, proteins and virus like particles (VLPs). Despite the many obstacles that impede the development of these vaccines, several studies involving VLP-based vaccine candidates have already generated promising results in preclinical studies (Murata et al., 2003; Jourdan et al., 2006; Elmowalid et al., 2007; Patient et al., 2009; Roy and Noad, 2009; Garrone et al., 2011; Vicente et al., 2011; Bellier and Klatzmann, 2013; Huret et al., 2013; Beaumont and Roingeard, 2015). This article will review HCV vaccine strategies based on homologous or heterologous HCV VLPs (**Table 1**), plasmid DNA and viral vectors encoding HCV-VLPs (**Table 2**) as well as various expression systems (**Table 3**) utilized in the production of HCV VLP vaccines.

**Table 1:** Homologous and recombinant Hepatitis C Virus (HCV) virus-like particle vaccines.

Viral structural components	Expression system	Cells	Route of administration	HCV antigen	Animal studies	Reference
HCV-core	Insect cells	Sf-9	Intraperitoneal and intramuscular	Core, E1, and E2	Mice and chimpanzees	Baumert et al., 1999; Lechmann et al., 2001; Murata et al., 2003; Elmowalid et al., 2007
	Mammalian	Huh-7	Subcutaneous	Core, E1, and E2	Mice	Kumar et al., 2016
	Yeast	*P. pastoris*	Subcutaneous	Core, E1 and E2	Rabbits	
HBcore	Bacteria	*E. coli* (GI698 cells)	Subcutaneous	Core (98 aa) and NS3 (155 aa)	Mice	Mihailova et al., 2006
		*E. coli* (K802 cells)	Subcutaneous	Core (60 aa) also HBV-S1 (27 aa)	Mice	Sominskaya et al., 2010
HBsAg	Mammalian	Huh-7, HEK293T	Subcutaneous	E2 HVR-1 (36 aa)	Mice	Netter et al., 2001, 2003; Vietheer et al., 2007
		CHO cells	Subcutaneous	E1, E2, and E1E2	Rabbits	Beaumont et al., 2013, 2016; Beaumont and Roingeard, 2015
PapMV CP	Bacteria	*E. coli*	Subcutaneous	E2	Mice	Denis et al., 2007
MLV Gag	Mammalian	HEK293T	Subcutaneous, intramuscular and intradermal	E1 and E1E2	Mice and macaques	Desjardins et al., 2009; Garrone et al., 2011; Huret et al., 2013
			Intradermal	NS3	Mice	Huret et al., 2013
TMV CP	Plant cells	*N. benthamiana*	Intranasal	E2 HVR-1 mimotope R9	Mice	Nemchinov et al., 2000


**Table 2:** Plasmid DNA and viral vectors expressing HCV virus-like particle vaccines.

Vector	Viral capsid components	Route of administration	HCV antigen	Animal studies	Reference
rVSV	HCV-core	Intravenous and intraperitoneal	Core, E1, and E2	Mice	Majid et al., 2006
Plasmid DNA	HCV-core	Intradermal and intramuscular	Core, E1, and E2	Mice	Murata et al., 2003
	MLV Gag	Intradermal	E1 and E2	Mice	Desjardins et al., 2009; Huret et al., 2013
			NS3	Mice	Huret et al., 2013


**Table 3:** Comparison of VLP expression system.

VLP expression system	Merits	Demerits
Bacteria	Less expensive; simplicity of expression; fast growth rate; high-level expression; genetic stability; simple process scale-up	Lack mammalian-like PTM; Poor ability on immunogenicity; Presence of host cell-derived contaminants
Yeast	Less expensive; high-density fermentation; modification of the expression protein; moderately rapid expression; support most protein folding and PTM	High mannose modification; some secretory proteins cannot get ideal results; enhanced safety precautions are required
Insect cells	Moderately rapid expression; support most protein folding and eukaryotic-type PTM of the expression protein; works well for non-enveloped and enveloped VLPs, free of mammalian pathogens	High cost; difficult to scale-up; incomplete modification of proteins; low-level expression, contamination of product with enveloped baculovirus particles; perform simpler *N*-glycosylation compared to mammalian cells
Mammalian cells	Perform appropriate complex mammalian-type PTMs; perform authentic assembly and folding of recombinant proteins; works well for non-enveloped and enveloped VLPs	High cost; difficult to scale-up; lengthy expression time; low yield; vulnerable to infection with mammalian pathogens
Plant cells	Rapid expression; highly scalable; less expensive; free of mammalian pathogens; support most protein folding and eukaryotic-type PTM	Low yield; technical and regulatory issues


## HCV VLP Vaccines in Pre-Clinical Studies

### HCV-Derived VLPs

Virus like particles are attractive candidates to elicit NAb responses as they structurally resemble and are much safer than the wild-type virus from which they are derived (Jourdan et al., 2006; Torresi et al., 2007; Roy and Noad, 2009). In addition to the hepatitis B virus (HBV) and HPV VLP vaccines, the VLP approach has been used to recently develop a chikungunya virus vaccine that was effective in non-human primates (Akahata et al., 2010). Self-assembled core-E1-E2 HCV-like particles (HCV-LPs) have been reported to generate HCV-VLPs with biophysical, ultrastructural and antigenic properties similar to those of the putative virion (Baumert et al., 1998, 1999). These HCV-LPs were generated in Sf9 cells from recombinant baculovirus encoding the HCV core, E1 and E2 proteins. Immunization with these recombinant HCV-LPs revealed that they can induce strong and broad polyclonal cellular and humoral immunity in mice (Baumert et al., 1999; Lechmann et al., 2001; Murata et al., 2003) as well as in chimpanzees (**Table 1**) (Elmowalid et al., 2007). The immunogenicity of HCV-LPs is strongly dependent on particle formation because vaccination with heat-denatured particles resulted in reduced antigen-specific T-cell and antibody responses (Lechmann et al., 2001). It is proposed that HCV-LP have the ability to interact specifically with dendritic cells (DCs) resulting in their activation and efficient antigen presentation, in contrast to denatured HCV-LPs (Murata et al., 2003; Barth et al., 2005).

Hepatitis C Virus-LPs that were produced in mammalian cells using an adenovirus-based system generated particles which were reported to resemble the native virions morphologically (Chua et al., 2012; Kumar et al., 2016). Vaccination with these adenovirus-derived HCV-LPs in combination with an anionic self-adjuvanting lipopeptide containing the Toll-like receptor (TLR) 2 agonist Pam_2_Cys (E_8_Pam_2_Cys) resulted in significant HCV-LP and E2-specific antibody responses mice (Chua et al., 2012). Up to three doses of non-adjuvanted or traditional alum-adjuvanted HCV-LPs were essential to reach similar HCV-LP- and E2-specific antibody titers induced with a single dose of HCV-LP+E_8_Pam_2_Cys. In addition, these antibodies neutralized the binding and uptake of VLPs by human hepatocellular carcinoma (Huh-7) cells and of cell culture derived HCV (HCVcc). Significant numbers of antigen-specific antibody secreting cells were also detected in the spleen of HCV-LP+E_8_Pam_2_Cys vaccinated mice, correlating with the previous results (Chua et al., 2012). Moreover, vaccination of human leukocyte antigen (HLA)-A2 transgenic mice with this vaccine generated higher HCV-LP-specific IFN-γ-mediated responses compared to non-adjuvanted HCV-LPs (Chua et al., 2012). In a heterologous prime-boost strategy, immunization with recombinant adenoviruses encoding the HCV structural proteins as a final booster, following priming with HCV-LPs, resulted in enhancement of both antibody and T-cell responses (Kumar et al., 2016). Additionally, the sera from immunized mice reduced the binding of VLPs and the JFH1 strain of HCV to Huh-7 cells demonstrating the presence of NAbs (Kumar et al., 2016). Even though different studies have provided evidence that HCV-derived VLPs are a promising vaccine strategy, capable of eliciting HCV-specific humoral and cellular immunity, additional studies are required to determine how best to induce highly protective, long-lasting immune responses.

### Recombinant HBV-Based VLPs Containing HCV Epitopes

Hepatitis B virus-derived VLPs have been evaluated as carriers able to present heterologous epitopes (Chackerian, 2007), including HCV-derived antigens. In one approach, chimeric VLPs were generated by fusing target antigens to viral capsid proteins (CPs) capable of self-assembling into VLPs (**Table 1**). The p24/p27 protein of HBV surface antigen (HBsAg-S) has been reported to self-assemble into potent, non-infectious, secreted subviral particles and has been used in commercial vaccines against hepatitis B for over three decades. HBsAg-S particles have been designed as carriers of foreign epitopes inserted at the N-or C-terminus or into the external hydrophilic loop of the HBsAg-S (Patient et al., 2009). HCV-specific epitopes derived from the E2 protein hypervariable region 1 (HVR1) were inserted into recombinant HBsAg VLPs which were secreted from transfected mammalian cells (Netter et al., 2001; Vietheer et al., 2007). These particles were recognized by human anti-HCV-positive serum containing anti-HVR1-1b antibodies, suggesting the antigenic structure of the HVR1 region expressed on the particles’ surface closely resembled the authentic structure (Netter et al., 2001). Subsequently specific antibody responses to HVR1 were efficiently induced (Netter et al., 2001; Vietheer et al., 2007). Furthermore, vaccination with a cocktail of HBsAg-S VLPs containing epitopes from either HCV-1a or -1b strains induced antibodies against both HVR1 epitopes that resulted in higher titers than those generated by immunization with the individual VLPs, suggesting a synergistic effect (Netter et al., 2001).

The insertion of the full-length HCV E1 and E2 envelope proteins into HBsAg VLPs was also shown to result in chimeric HBV-HCV particles similar in size and shape to the wild-type HBsAg subviral particles (Patient et al., 2009). Immunization of rabbits with these HBV-HCV VLPs resulted in high titers of cross-NAbs with the ability to neutralize various HCV genotypes (Beaumont et al., 2013). Recently, the immunogenicity of the chimeric HBsAg VLPs expressing E1 and E2 proteins as separate immunogens were compared with HBsAg VLPs bearing an E1E2 heterodimer (Beaumont et al., 2016). The E1- and E2- specific humoral responses induced in animals vaccinated with HBsAg VLPs expressing E1E2 in their heterodimer conformation, were significantly reduced compared to the responses in rabbits immunized with HBsAg VLPs expressing E1 and E2 separately (Beaumont et al., 2016). Additionally, the E1- and E2-specific antibodies showed increased cross-neutralization of various genotypes of heterologous HCV strains. These results confirmed previous observations (Garrone et al., 2011), that the E1 and E2 heterodimer is less immunogenic, possibly due to the conformational folding of E1 and E2 at the surface of the HBsAg particles contributing to the masking of certain immunodominant epitopes. Furthermore, although the folding of E1–E2 complex on the surface of the HBsAg particles was demonstrated, the structure of the E1E2 dimers on these particles might not be representative of dimers on the surface of native HCV virions. Nonetheless, these particles also generated significant levels of HBs-specific antibodies, further supporting the development of a bivalent HBV-HCV prophylactic vaccine capable of preventing primary infection with either of these two viruses (Beaumont et al., 2016). Administration of chimeric HBsAg-S/HCV VLP demonstrated that mice (Netter et al., 2003) and rabbits (Beaumont and Roingeard, 2015) with pre-existing anti-HBsAg antibodies successfully generated anti-HCV antibodies, therefore these chimeric particles can be used in populations with pre-existing immunity to HBV as a strategy to induce protective immunity to HCV. The encouraging results obtained so far support the development of HBV-derived VLP vaccines. However, additional studies are needed to assess the immunogenicity of these chimeric HBV/HCV particles in a larger animal model and also to develop means to further increase the broadly neutralizing characteristics of the resultant NAbs.

The HBV core protein (HBc) naturally self assembles into dimers and subsequently into VLPs and has been used as a VLP carrier for over three decades (Clarke et al., 1987; Borisova et al., 1993). Insertion of foreign sequences into the HBc gene does not impede particle formation making HBc suitable for the development of a VLP vaccine displaying a foreign antigen (Ulrich et al., 1998; Sominskaya et al., 2010). Segments of HCV genes encoding the core (98 aa) or NS3 (155 aa) proteins, containing B- and T-cell epitopes, were fused to the 3′ end of the HBc gene to generate chimeric HBc/HCV VLPs (Mihailova et al., 2006). These two proteins were chosen as immunogens because the induction of broad CD4+ and CD8+ T-cell responses to various HCV proteins, including core and NS3 is typically associated with resolution and control of HCV infection (Neumann-Haefelin et al., 2005). Administration of the HBc/HCV core particles induced moderately low antibody levels and proliferative responses of T-cell to HCV core epitopes, while vaccination with the HBc/HCV NS3 particles induced significant levels of anti-NS3 antibodies with no proliferative responses to HCV epitopes (Mihailova et al., 2006). However, antibodies to core and NS3 are believed to have no prophylactic value and are generally not considered to be protective in natural HCV infection. Furthermore, variations in the immune responses to the chimeric particles might be caused by alternative presentation of inserted HCV sequences in the context of the chimeric particles (Mihailova et al., 2006). Similarly, chimeric VLPs carrying a virus-neutralizing HBV pre-S1 epitope and a highly conserved N-terminal HCV core epitope at the C terminus of the truncated HBc protein (HBc-pre-S1-HCVcore) have been successfully generated (Sominskaya et al., 2010). Vaccination with these particles induced high T cell immunity similar to that induced by the monovalent HBc-HCV core particles, but induced low antibody responses to HCV core epitopes. It was argued that the core epitopes located at the C terminus were not exposed on the surface of the VLP and therefore were not able to induce strong antibody responses (Sominskaya et al., 2010). This further highlights the importance of the position of inserted epitopes within HBc in terms of the particle’s humoral immunogenicity. Insertion of larger amino acid sequences would be beneficial for the development and production of a multivalent HCV vaccine carrying a larger number of epitopes coupled to one carrier molecule and facilitate proper folding of conformational epitopes which is important for the generation of NAbs. However, inserting larger inserts within the HBc particles might affect the ability of the chimeric particles to drive insert-specific immune responses. Various studies have reported contradictory results regarding the maximum insertion capacity of HBc without affecting immunogenicity to the insert antigen and HBc’s ability to successfully form particles. A maximum of 90 aa of human immunodeficiency virus (Kalams et al., 2013). Gag protein was reported to be tolerated while fusion of 317, 189, or 100 aa of the Gag protein downstream of aa 144 prevented self-assembly of the chimeric HBc particles (Ulrich et al., 1992). Yoshikawa et al. (1993), on the other hand, reported successful formation of HBc particles carrying a 720 aa segment composed of four copies of an 180 aa sequence derived from HCV core protein. However, the immunogenicity of these particles was not assessed in an *in vivo* model.

### Recombinant Retrovirus-Based HCV VLPs

Complete glycoproteins can also be displayed in their native conformation on the surface of retroviral particles by a process known as pseudotyping. Infectious HCVpp which are engineered by pseudotyping HCV glycoproteins onto murine leukemia virus (MLV)-Gag retroviral core particles have been used to comprehensively study the early events of HCV infection, as well as the role of putative HCV receptors (Bartosch et al., 2003b). These particles are also commonly used to detect and measure anti-HCV NAb in HCV patients (Bartosch et al., 2003a; Drummer et al., 2003). HCV-pseudotyped retrovirus-derived VLPs devoid of a genome (HCV-retro VLPs) have been proposed as a vaccine platform. Studies of the molecular structure of these pseudoparticles described HCVpp and HCV-retro VLPs as regular 100 nm spherical structures comprised of the dense retroviral nucleocapsid surrounded by a lipid bilayer (Bonnafous et al., 2010; Garrone et al., 2011). Following priming with HCV-recombinant viral vectors, VLPs pseudotyped with E2 and/or E1 HCV glycoproteins generated significant anti-E2 and/or anti-E1 antibody titers in mice and macaques (Garrone et al., 2011). This highlighted the difficulty of inducing an anti-E1 antibody response, consistent with the findings that these antibodies are generally detected at low levels in patients (Leroux-Roels et al., 1996; Pestka et al., 2007). Of the regimens examined, only the VLPs induced significant levels of anti-E1 antibodies, which were generated provided that E1 was dissociated from E2. More importantly, boosting with the retrovirus-derived VLPs in a heterologous prime-boost combination with plasmo-retroVLP raised NAbs against HCV genotype 1a which cross-neutralized five other genotypes/strains (1b, 2a, 2b, 4, and 5) (Huret et al., 2013). No challenge experiments were conducted to assess the anti-HCV potency of these HCV-retro VLPs. Overall, retrovirus-derived VLPs pseudotyped with an assortment of virus envelopes, represent a versatile and efficient platform for vaccination not only against HCV but also against major infectious diseases such HIV/AIDS, yellow fever and dengue fever. However, despite these encouraging results, the use of animal retroviral particles has not been validated as a safe or effective preventative vaccine in humans, and scaling up the manufacturing process may present a limiting factor for the development of recombinant retrovirus-based HCV VLP vaccines.

### Recombinant Vesicular Stomatitis Virus Vectors Expressing HCV VLPs

Recombinant vesicular stomatitis virus (rVSV) has been shown to be an effective expression vector in a number of different vaccine strategies (**Table 2**) (Schnell et al., 1996; Kretzschmar et al., 1997). There are several advantages of using rVSV encoding foreign viral proteins as a vaccine candidate. The virus has a low seroprevalence in the community and is not pathogenic in humans; the simple genome has only five genes (N, P, M, G, and L) and it does not undergo reassortment or integration (Johnson et al., 1966; Fields and Hawkins, 1967; Ezelle et al., 2002). Recombinant VSV has been evaluated as a candidate for HCV vaccination by encoding HCV core, E1 and E2 proteins (rVSV-C/E1/E2) which can self-assemble into HCV VLPs (VSV-HCV-VLPs) in baby hamster kidney fibroblasts (BHK-21 cells) (Ezelle et al., 2002). These VSV-HCV-VLPs showed similar ultrastructural properties to HCV virions (Ezelle et al., 2002). Vaccination with these particles induced core-, E1- and E2-specific cell mediated responses as well as anti-E2 antibody responses (Ezelle et al., 2002). However, it was argued that these particles may represent the endogenous viruses of BHK-21 cells known as intracisternal R-type particles, rather than the complete budded HCV-like particles (Blanchard et al., 2003). Subsequently it was demonstrated that the expression of HCV E1 and E2 by replication-competent and -defective (G-protein-deleted) VSV vectors resulted in correctly folded E1/E2 heterodimers (Majid et al., 2006). Nevertheless, this study only assessed the expression of the envelope proteins but did not provide a detailed characterization of the formed VSV-HCV-VLPs. Immunization with rVSVΔG-C/E1/E2 resulted in significant T cell responses to core, E1 and E2, along with anti-core and anti-E2 antibody responses (Ezelle et al., 2002; Majid et al., 2006). Neutralizing antibody responses were not assessed. However, immunization with rVSVΔG-C/E1/E2 protected mice against the formation of tumors expressing HCV E2 (CT26-hghE2t) and showed CT26-hghE2t-specific as well as E2-specific T-cell responses. Recombinant VSV expressing HCV structural proteins represent a versatile tool in vaccine development, which cannot only be used as a vector for the production of HCV VLPs in mammalian cells but also as a vaccine candidate itself. The recent effectiveness of an Ebola vaccine candidate rVSV-ZEBOV (Regules et al., 2017), comprising a live-attenuated VSV encoding the Ebola virus glycoprotein, in preventing Ebola virus disease in recently diagnosed patients, their contacts and contacts’ contacts (ElSherif et al., 2017; Halperin et al., 2017; Henao-Restrepo et al., 2017) provides hope for the development of a VLP-based HCV vaccine using this strategy. However, further studies are required to better characterize and improve the production VSV-HCV-VLPs. The protective immunity generated by rVSVs encoding HCV core, E1 and E2 also needs to be evaluated before these particles can be used as a vaccine against HCV infection.

### DNA Vaccines Encoding Recombinant Retrovirus-Based HCV VLPs

DNA plasmids generating recombinant retro-VLPs (plasmo-retroVLPs) (Bellier et al., 2006, 2009; Desjardins et al., 2009) represent a HCV genetic vaccine circumventing the *in vitro* production and purification of retro-VLPs. These plasmo-retroVLPs have been engineered to produce *in situ* retroVLPs pseudotyped with E1 and E2 proteins after *in vivo* delivery of the DNA (**Table 2**). By doing so, this strategy combines the benefits of DNA vaccines such as the ease of development, low-cost large-scale production and stability with the immuno-stimulatory properties of VLPs, while circumventing the *in vitro* production of VLP vaccines. It has been shown that administration of plasmo-retroVLPs result in the induction of significantly higher antigen-specific responses and antiviral immune protection than plasmids that were mutated to prevent plasmo-retroVLP assembly (Bellier et al., 2006, 2009). It was also shown in heterologous prime-boost immunization strategies, that HCV-plasmo-retroVLPs were superior immunogens for boosting cellular and humoral immune responses in primed animals than plasmids unable to form E1/E2-pseudotyped retroVLPs (Bellier et al., 2009; Desjardins et al., 2009). A combination of two plasmo-retroVLPs vaccinations followed by a boost with *ex vivo* produced VLPs was shown to induce HCV-specific cell-mediated response and NAbs (Huret et al., 2013). Furthermore, vaccination with a cocktail of plasmo-retroVLPs pseudotyped with E1E2 from HCV genotypes 1a, 1b, 2b, 3a, 4c, and 5, and/or displaying NS3 antigen in CPs resulted in immune responses against the five HCV genotypes (Huret et al., 2013). A major advantage of plasmo-retroVLPs is that they can be re-administered for sequential vaccinations without the concern of pre-existing vector- associated immunity (Bellier et al., 2009). DNA-based vaccines, however, generally induce relatively weak immune responses to antigens and are therefore regarded as inferior compared to traditional vaccines such as subunit vaccines. As such, even the above strategy required boost immunizations with purified VLPs for maximum activity. The delivery route and/or method of DNA-based vaccines is also a critical factor in determining vaccination outcome. VSV-G specific plasmo-retroVLPs have been shown to induce higher levels of VSV-G-specific humoral and cell mediated immune responses following administration by needle-free intradermal (i.d.) injection compared to immunization with a gene gun (Bellier et al., 2009). Additionally, needle-free i.d. co-injection of cytokine genes or CpG sequences significantly increased VSV-G-specific NAbs levels (Bellier et al., 2009). Therefore, attempts should be directed toward the optimization of the delivery methods of DNA-based VLP vaccines and the addition or co-delivery of novel genetic adjuvants for maximum immune stimulation. Together, these results show that the plasmo-retroVLPs is a flexible platform to induce humoral and cellular immunity after homologous or heterologous prime-boost immunization and with further improvements, this strategy could be used as a promising approach to generate enhanced immune responses against HCV.

### Recombinant Papaya Mosaic Virus-Based HCV VLPs

Virus like particles derived from plant viruses have also emerged as a promising strategy for the development of viral vaccines. The papaya mosaic virus (PapMV) CP has triggered much interest as an epitope presentation system. Several hundred units of the PapMV CP have been reported to assemble in an organized and repetitive manner into particles ranging from 60 to 100 nm in length following expression in *Escherichia coli* (Tremblay et al., 2006). A recombinant multimeric PapMV vaccine platform expressing a HCV-E2 peptide fused to the C-terminal PapMV CP was shown to trigger a strong humoral immune response in mice lasting more than 120 days against the CP and the E2 epitope (**Table 1**) (Denis et al., 2007). Notably, the same platform was less effective when inoculated as a monomeric protein, confirming the observation that immunogenicity is strongly dependent on the repetitive organization of the antigen (Denis et al., 2007). This VLP platform faces challenges which might limit its clinical development as vaccine, including the presence of bacterial RNA used a scaffold for PapMV CP self-assembly (Tremblay et al., 2006), as well as the possibility that antigen fusion might alter the antigen conformation or interfere with the protein assembly and VLP formation.

## HCV VLP Production Systems

Many expression systems for the production of VLPs are currently in use, including various species of yeast such as *Saccharomyces cerevisiae* (*S. cerevisiae*) and *Pichia pastoris* (*P. pastoris*), bacteria (mainly *E. coli*), the baculovirus expression vector/insect cell system (BEVS/IC), and various mammalian cell lines, as well as various plants (tobacco, lettuce leaves) (**Table 3**). The production system used depends on the need to retain more consistent original VLP formation and post translational modifications (PTMs, e.g., glycosylation and phosphorylation) while significantly reducing production times and costs. All production systems have advantages and drawbacks as discussed briefly.

### Bacteria and Yeast

Bacterial systems are the most commonly used system to manufacture recombinant proteins and have been used to produce non-enveloped HCV VLPs. *E. coli*-derived HCV core proteins self-assembled *in vitro* into particles of approximately 60 nm in diameter (Kunkel et al., 2001). Lorenzo et al. (2001) on the other hand, produced VLPs in *E. coli* following expression of truncated HCV core protein consisting of the first 120 amino acids. Similarly, the recombinant HCV-E2 PapMV CP VLPs were also produced following expression in *E. coli* (Denis et al., 2007; Rioux et al., 2012). However, several factors including the lack of a mammalian-like PTM system means that this platform is not preferred for VLP production (**Table 3**). Additionally, the downstream processing of the VLPs is compromised by the presence host cell derived toxins, heat shock proteins or chaperone proteins, increasing the final production cost (Zhang et al., 1998; Lai and Middelberg, 2002).

Yeast is another well-established platform used for recombinant protein expression and VLP production. Four licensed VLP-based vaccines, Engerix-B^®^, Hepavax-Gene^®^, Recombivax HB^®^ and Gardasil^®^ (Merck and Co), are manufactured using this system. The entire HCV core protein expressed in methylotrophic *P. pastoris* formed particles with similar structural properties as the native mature HCV nucleocapsid particles, and were successfully recognized by serum from a persistently infected HCV patient (Acosta-Rivero et al., 2001, 2004). More recently, VLPs of 70 nm in diameter were generated following expression of recombinant HCV coreE1E2 proteins in *P. pastoris* and were able to efficiently induce anti-CoreE1E2 antibodies in rabbits (Fazlalipour et al., 2015). However, concerns about appropriate protein processing, protein folding, and post translational modification, may direct the choice toward an alternative production system (Acosta-Rivero et al., 2004; Kushnir et al., 2012).

### Mammalian Cells

Mammalian cell expression systems are favored because they are capable of performing appropriate complex mammalian-type PTMs and authentic assembly of recombinant proteins (Chua et al., 2012; Earnest-Silveira et al., 2016b; Kumar et al., 2016). Various HCV VLPs vaccine candidates have been successfully produced in mammalian cells including Chinese hamster ovary (CHO), Huh-7cells, and human embryonic kidney (HEK) 293T cells (**Table 1**). Immunization with recombinant HCV envelope glycoproteins produced in mammalian cells protected chimpanzees more effectively compared to those produced in yeast or insect cells after challenge with a homologous HCV isolate (Choo et al., 1994). Similarly, mammalian cell-derived recombinant envelope proteins have been reported to bind strongly to human cells compared to those produced in yeast or insect cells (Rosa et al., 1996). More recently VLPs produced in mammalian cells were found to be more immunogenic in a VLP prime/Rec-Ad-C-E1-E2 boost based heterologous strategy, when compared to VLPs produced using the baculovirus-insect cell system (Kumar et al., 2016). To avoid the deleterious effects of cesium chloride (CsCl), the use of iodixanol ultracentrifugation and stirred cell ultrafiltration resulted in the production of large quantities of HCV VLPs from various genotypes as a strategy for the manufacture of a quadrivalent mammalian cell derived HCV VLP vaccine (Earnest-Silveira et al., 2016a,b).

### Plant Cells

Plant production platforms have many advantages compared to mammalian cells (**Table 3**). Plant-derived VLPs offer a new approach for oral delivery of vaccines. Plant expression systems are highly scalable, economical and free of mammalian pathogens. Additionally, plant production systems possess eukaryotic-like protein folding and PTMs similar to mammalian cells (Mett et al., 2008; Yusibov and Rabindran, 2008; Kushnir et al., 2012). Plant expression systems are suitable for the production of both non-enveloped and enveloped VLPs (D’Aoust et al., 2008; Santi et al., 2008). A tobacco mosaic virus (TMV)-derived vector encoding a HVR1peptide (containing a HCV neutralizing epitope) fused to the C-terminus of the B subunit of cholera toxin (CTB) was used to infect tobacco plants (*Nicotiana benthamiana*) (Nemchinov et al., 2000; Rybicki, 2014). Infected plants successfully generated TMV particles expressing HVR1/CTB (Nemchinov et al., 2000). The plant-derived HVR1/CTB reacted with anti-HRV1 monoclonal antibodies and sera from HCV-positive individuals infected with four of the major genotypes of HCV. Inoculating mice with plant extract resulted in the induction of both anti-CTB and anti-HVR1 antibodies capable of specifically binding to HCV VLPs (Nemchinov et al., 2000). A plant-derived HCV vaccine can reduce manufacturing costs associated with the production of conventional vaccines and the vaccine can be administered in edible plant part this would particularly helpful in reducing incidence of HCV in the developing world. However, to date no plant-derived VLPs have been licensed for human use. In addition, the dosage required for effective vaccination might vary depending on the plants used, ripeness of the fruit and the quantity of food consumed. Therefore, administration of edible vaccines requires to be standardized to evaluate the dosage requirement for effective vaccination. Also, certain plants and fruits are generally not eaten raw and cooking would result in denaturation of the protein and a reduction in immunogenicity to the vaccine. Most plants and fruits are susceptible to microbial infestation and this would affect the stability of the vaccine.

### Insect Cells

The baculovirus-insect cell expression system has also been widely used for VLP production (van Oers et al., 2015). Insect cells have been employed to express several VLP-based vaccines, notably one of the current HPV vaccines, Cervarix^®^. The baculovirus–insect cell system can be used to manufacture non-enveloped and enveloped VLPs (**Table 3**) and various HCV VLPs have been successfully produced using this system (**Table 1**) (Baumert et al., 1999; Wellnitz et al., 2002; Choi et al., 2004; Zhao et al., 2004). The insect cell system can perform eukaryotic-type PTMs, support high expression levels of foreign proteins with the advantage of lacking mammalian pathogens (Roy and Noad, 2008; Kushnir et al., 2012). Expression of core, E1 and E2 proteins in the recombinant baculovirus system resulted in the formation of recombinant virus particles (Baumert et al., 1998, 1999, 2000) capable of inducing antibodies and cell-mediated immune responses after immunization in rabbits. A major disadvantage of this system is contamination of the product with co-produced enveloped baculovirus particles (Buonaguro et al., 2006; Palomares and Ramirez, 2009), which require the development of more complex and stringent VLP purification systems. Another limitation of this system is that insect cells perform simpler *N*-glycosylation compared to mammalian cells, which is troublesome in vaccine developments as correct glycosylation is often necessary for optimum immunogenicity of the vaccine antigen (Kost et al., 2005; Orlova et al., 2015).

## Downstream Processing of VLP Vaccines

The purity, potency and consistency of the particles and elimination of host cell and culture media contaminants are crucial for the downstream processing of VLP-based vaccines. In addition, the purification methods should be robust, cost-effective, scalable and preferably applicable to a wide variety of VLPs.

Virus like particles are purified using methods originally developed for the purification of viruses by which particles are purified based on their size and density using ultracentrifugation through sucrose or CsCl gradients (Vicente et al., 2011). However, ultracentrifugation-based methods are not practical for large scale vaccine development as they are non-scalable, tedious and highly labor intensive. Furthermore, use of CsCl is problematic due to its toxicity, reduced infectivity of viruses and particle deformation (Burova and Ioffe, 2005). Due to these limitations, there is currently a trend moving away from traditional VLP purification methods toward more scalable sophisticated techniques such as chromatography (Morenweiser, 2005; Vicente et al., 2011). Here, crude lysate is initially clarified by low-speed centrifugation or tangential flow filtration prior to chromatography (ion exchange, affinity, or size-exclusion). Chromatography provides a convenient and practical intermediate step for capturing and concentrating VLPs from cellular and media contaminants (Morenweiser, 2005).

## Conclusion

The path to a HCV vaccine has been fraught with difficulties mainly because of the virulent nature of the virus and its ability to evade the immune responses due to its heterogeneity. However, advances in technology and our understanding of the natural course of HCV infection, the pathogenetic mechanisms, and the immunological markers which correlate with resolution of infection or protection, provide useful information for vaccine design. VLP vaccines have many favorable immunological characteristics making them promising HCV vaccine candidates. Currently, no VLP-based HCV vaccines have progressed to human clinical trials. However, VLP-based vaccines for the prevention of HBV and HPV infections have already been licensed, supporting the development of VLP-based vaccines for HCV. The HCV VLP-based vaccine candidates that have been developed so far have generated promising data in pre-clinical animal studies. It is widely known that several factors, including the expression system, will affect the quality and production yields of viral particles. Several expression systems have been effectively used to produce VLP vaccine candidate. Some VLP-based HCV vaccine candidates such as chimeric HBV/HCV VLPs can be produced using already established production systems such as bacterial and yeast. However, these particles can be contaminated with residual host cell factors such as lipids, nucleic acids and proteins that may stimulate innate immune responses and/or reduce the specific adaptive immune response and therefore may have a significant impact on VLP-based vaccine development that could present a bottleneck in their development. VLPs produced using mammalian cells offer an alternative system which supports appropriate complex mammalian-type PTMs and performs authentic assembly and folding of recombinant proteins and therefore generates VLPs that are very similar to the authentic HCV virion. However, difficulties in scaling up this system and high-production costs might direct the choice toward more cost effective alternative production system. Genetic vaccines that express HCV-recombinant VLPs, on the other hand are attractive candidates as the production of high quality plasmid DNA is simple and inexpensive to scale-up and could further advance the development of novel VLP-based strategies. Ultimately, it is hoped that newly developed VLPs can elicit specific and strong responses to HCV to prevent and contain infection. These encouraging results from pre-clinical animal studies suggest that it should be possible to develop such a vaccine although this may yet be some way off.

## Author Contributions

MM conceived the initial draft of the manuscript. DW, JT, EG, and BG-B revised many parts of the manuscript, and contributed to finalize the manuscript.

## Conflict of Interest Statement

The authors declare that the research was conducted in the absence of any commercial or financial relationships that could be construed as a potential conflict of interest.
